# Chitosan-Integrated Curcumin–Graphene Oxide/Copper Oxide Hybrid Nanocomposites for Antibacterial and Cytotoxicity Applications

**DOI:** 10.3390/antibiotics13070620

**Published:** 2024-07-03

**Authors:** Anandhavelu Sanmugam, Logesh Kumar Sellappan, Abbishek Sridharan, Swathy Manoharan, Ananda Babu Sairam, Abdulrahman I. Almansour, Subha Veerasundaram, Hyun-Seok Kim, Dhanasekaran Vikraman

**Affiliations:** 1 Department of Applied Chemistry, Sri Venkateswara College of Engineering, Sriperumbudur 602117, India; anandhavelu@svce.ac.in (A.S.); anandababu@svce.ac.in (A.B.S.); 2 Department of Chemical Engineering, Indian Institute of Technology Hyderabad, Kandi 502285, India; ch24ipdf15@iith.ac.in; 3 Materials Engineering, RWTH Aachen University, 52062 Aachen, Germany; abbishek.sridharan@rwth-aachen.de; 4 Department of Biomedical Engineering, KPR Institute of Engineering and Technology, Coimbatore 641407, India; swathy.m@kpriet.ac.in; 5 Department of Chemistry, College of Science, King Saud University, P.O. Box 2455, Riyadh 11451, Saudi Arabia; almansor@ksu.edu.sa; 6 Department of Chemistry, R.M.D. Engineering College, Tiruvallur 601206, India; subha.snh@rmd.ac.in; 7 Division of Electronics and Electrical Engineering, Dongguk University-Seoul, Seoul 04620, Republic of Korea; hyunseokk@dongguk.edu

**Keywords:** chitosan, graphene oxide, curcumin, CuO, antibacterial, cytotoxicity

## Abstract

This study deals with the facile synthesis of a single-pot chemical technique for chitosan–curcumin (CUR)-based hybrid nanocomposites with nanostructured graphene oxide (GO) and copper oxide (CuO) as the antibacterial and cytotoxic drugs. The physicochemical properties of synthesized hybrid nanocomposites such as CS-GO, CS-CuO, CS-CUR-GO, and CS-CUR-GO/CuO were confirmed with various advanced tools. Moreover, the in vitro drug release profile of the CS-CUR-GO/CuO nanocomposite exhibited sustained and controlled release during different time intervals. Also, the antibacterial activity of the CS-CUR-GO/CuO hybrid nanocomposite presented the maximum bactericidal effect against *Staphylococcus aureus* and *Escherichia coli* pathogens. The hybrid nanocomposites revealed improved cytotoxicity behaviour against cultured mouse fibroblast cells (L929) via cell adhesion, DNA damage, and proliferation. Thus, the chitosan-based hybrid nanocomposites offer rich surface area, biocompatibility, high oxidative stress, and bacterial cell disruption functionalities as a potential candidate for antibacterial and cytotoxicity applications.

## 1. Introduction

Chitosan (CS) is a positively charged linear polysaccharide randomly arranged as β-(1-4)-D-glucosamine and N-acetyl-D-Glucosamine [1,2]. CS is a natural biopolymer derived from chitin by the deamination process [3]. Owing to the presence of surface amino groups, CS contributes positive zeta potential generation [4,5]. CS is the most commonly available polysaccharide used in various applications, including applications for food packaging, medicine, cosmetics, textiles, water treatment, and agriculture, due to its superior characteristics of biodegradability, biocompatibility, swelling, nontoxicity, antibacterial properties, cell adhesion, and cell proliferation [6]. The cationic behaviour and hydrophilic nature of CS support both physical and chemical interaction during complex matric formations with nanoparticles, drugs, co-polymers, and crosslinkers [7]. CS can easily proliferate fibroblast and keratinocyte cells in the creation of an extracellular matrix with the presence of active amino acids [8,9]. Also, CS biopolymer enhances antibacterial activity by inhibiting bacterial cell growth; especially when treated with *Staphylococcus aureus* (*S. aureus*) pathogens, CS promotes structural changes, damages the surface structure of cells, and initiates bacterial death [10,11].

Graphene oxide (GO) is a carbon-related monolayer system with desired characteristics such as optical, thermal, mechanical, biological, and chemical properties. GO-based nanocomposites attract various biomedical applications, including bioimaging, cancer therapy, drug delivery, wound dressing, tissue regeneration, biosensors, and antibacterial activity [12,13]. However, the active functional groups like hydroxyl, carbonyl, phenol, quinone, and epoxide on the surface form a basal plane that possesses electron mobility, charge transfer, biocompatibility, high surface area, and reactive oxygen species (ROS) formation [14]. Although GO has adsorption, hydrophilic, and enhanced photocatalytic properties, the chemically reduced graphene oxide has minimal cytotoxic effects compared to GO [15]. As GO-based nanocomposites are versatile, nanocarriers can be easily encapsulated for drug delivery, damage to cell membranes, charge transfer across nanocomposites, and inhibiting DNA replication [16]. Curcumin (CUR) is a bi-phenolic compound, [1,7-bis(4-hydroxy-3-methoxy phenyl)-1,6-heptadiene-3,5-dione], found in turmeric [17]. It is often used as a colouring agent but is highly antibacterial and anticancerous in biomedical applications [18]. It has been used for hundreds of years to treat various ailments [18]. In addition, CUR combines with other nanocomposites and scavenges free radicals, assists drug loading, provides a large surface area, induces pharmacological effects to decrease inflammation, and assists cell proliferation and cell adhesion functionalities at damaged tissue sites [19,20]. However, the previous literature ascribed that curcumin can easily integrate with CS, GO, and other metal oxides [21]. CUR is a pleiotropic molecule that regulates cellular signal pathways during genetic mutations and protein regulation. Curcumin can break the penetrability and reliability of the membranes of bacterial cells, which leads to cell fatality [22]. Further, the lipophilic structure of curcumin permits it to be inserted into liposome bi-layers, thereby enhancing their permeability [23]. Curcumin can stimulate the permeabilization of Gram-positive and -negative cell walls, which is directly related to the bactericidal carnage effect of curcumin [24]. Also, the bioavailability of CUR increases the incorporation of nanocomposites (which favours nontoxicity and high stability), exhibits adsorption, assists tissue repair, eliminates drug-resistant microorganisms, induces cytotoxicity, and promotes cell growth [25,26]. However, poor absorption, rapid metabolism, and rapid excretion are believed to be the main reasons for curcumin’s low bioavailability [27].

Currently, the design and development of metal- and metal oxide-based nanocomposites enhance various biomedical uses like biosensing, bioimaging, tissue engineering, drug delivery, wound healing, bone regeneration, implant coatings, and antibacterial applications [28,29]. The incorporation of nanoparticles is an effective route to enhance antibacterial properties [30]. The most prominent metals and metal oxides for antibacterial studies include gold, silver, copper, zinc, cerium, titanium, iron, and selenium [31,32]. Metals/metal oxides can enter into the microorganisms’ cells owing to the explicit kinetics for cell deactivation. The key factor of the biocidal kinetics is enacted by freed metal ions distributed from the nanoparticles’ surface and reactive oxygen species (ROS) producing oxidative stress [33,34]. Among these, copper oxide (CuO) nanostructures are effective catalysts yielding high thermal stability and drug encapsulation and are relatively cheaper than silver and gold composites [35,36]. Moreover, CuO is widely used due to its reinforcing capacity, improved mechanical properties, biocompatibility, assisted wound reconstruction, decreased inflammation, and cell adhesion [37]. Previous studies mentioned CuO nanoparticles can easily interact with biological systems at the cellular level to trigger different responses and functions [38,39]. The antibacterial effect of CuO has been associated with a rapid weakening of the integrity of cell membranes and ROS creation [40]. The redox nature of Cu(i) and Cu(ii) create superoxide groups that impact the deprivation of biomolecules [38]. Although pure metal oxides have a wide-ranging spectrum of pharmacological behaviours, the appropriate therapeutic uses and their bio-applicability after administration are very low. 

Based on detailed studies of the literature, herein, we developed CS-GO, CS-CuO, CS-CUR-GO, and CS-CUR-GO/CuO composites. The advanced characterization studies authoritatively confirmed the formation of the nanocomposites. The drug delivery characteristics were suggestively studied for the prepared nanocomposites. Further, antibacterial characteristics were evaluated versus *Escherichia coli* (*E. coli*) and *S. aureus* bacteria. Detailed investigation of cytotoxicity, in vitro, against L929 fibroblast cell lines was extended by the prepared nanocomposites.

## 2. Results and Discussion

**Figure 1:**
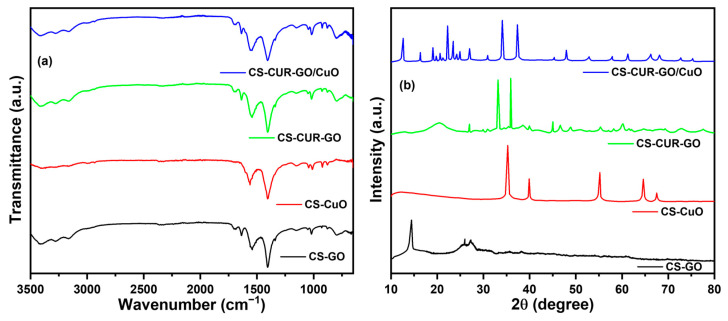
(**a**) FT-IR and (**b**) XRD spectra of CS-GO, CS-CuO, CS-CUR-GO, and CS-CUR-GO/CuO composites.

The functional groups of the CS-GO, CS-CuO, CS-CUR-GO, and CS-CUR-GO/CuO composites were examined using FT-IR spectroscopy. Figure 1a demonstrates the FT-IR spectral results of the prepared CS-based hybrid composites. For CS-GO, the OH functional group stretching vibration peaks are exhibited at 3450–3100 cm^−1^ [41]. The C=O and C=C vibrations are exhibited at 1704 and 1638 cm^−1^, respectively, which strongly ascertain the GO formation [42,43,44]. The doubling group NH_2_ peak around 1550 cm^−1^ confirms the CS exhibited in the resulting composites. An amide III peak is exhibited at 1364 cm^−1^ for the CS-GO composites [45]. The peaks at around 1160 cm^−1^ and 1020 cm^−1^ are credited to the stretching C–OH and stretching C–O–C bands, respectively [45]. The weak band at 877 cm^−1^ is credited to the presence of glycosidic linkage in the CS-CuO. The peak around 650 cm^−1^ authenticates the creation of CuO stretching [46]. For the CS-CUR-GO and CS-CUR-GO/CuO structures, the accumulative peaks confirm the formulation of the nanocomposites.

Figure 1b shows the X-ray diffraction (XRD) peaks of the CS-based hybrid nanocomposites. From the XRD profiles, the angles of diffraction and their intensities for the hybrids agree well with each other. The GO-related XRD peaks are observed at around 14° due to the (002) lattice plane for CS-GO, which is consistent with the previous reports [47,48]. Further, the CS-related 101 and 130 lattice planes are exhibited for CS-GO at 2θ 25.3° and 27.1°, respectively [49]. For CS-CuO, CS-related peaks are diminished due to the high crystalline CuO peak presence. Further, CuO (002), (111), (020), (−311), and (220) lattice planes are exhibited for the CS-CuO nanocomposites [50]. CUR produces various crystalline peaks due to poly-orientation, as consistent with previous reports [18,51]. Furthermore, the CS-CUR-GO/CuO nanocomposite shows highly intense peaks owing to the integration of GO and CuO. Furthermore, all the diffraction lines show sharper peaks which reveal the strong crystallinity and improvement of the crystallite size of the nanocomposites. The microstructural properties of the hybrid nanocomposites were also calculated using Scherer’s formula [52,53]. The estimated average crystallite size of the CS-CUR-GO and CS-CUR-GO/CuO nanocomposites are 29 and 24 nm, respectively. Supplementary Materials Figure S1 shows the XRD profiles of pure CS and CUR, which establishes the semi-crystalline nature of the prepared pure structures. The derived FT-IR and XRD outcomes confirm the nanocomposite formation.

**Figure 2:**
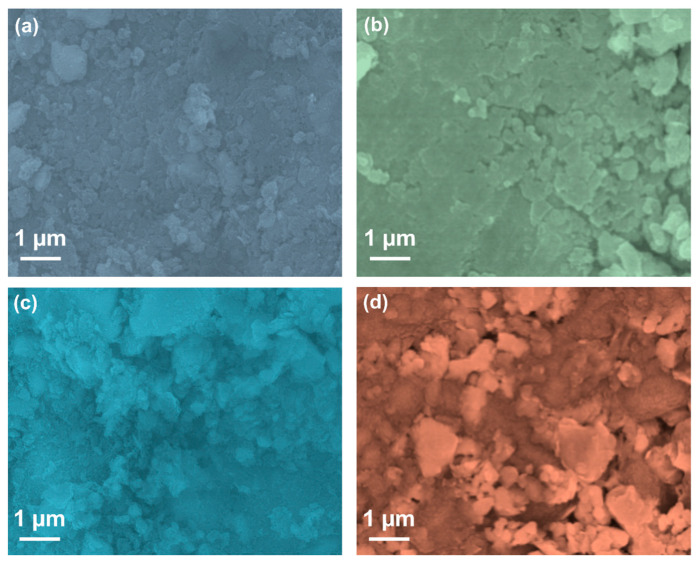
SEM analysis of (**a**) CS-GO, (**b**) CS-CuO, (**c**) CS-CUR-GO, and (**d**) CS-CUR-GO/CuO nanocomposites.

To elaborate on the surface properties of the prepared composites, scanning electron microscopy (SEM) analysis was performed. Figure 2a–d depict the SEM images of the CS-GO, CS-CuO, CS-CUR-GO, and CS-CUR-GO/CuO nanocomposites. The CS-GO composite produces nano-sized grain bunches comprised of various shapes of fibre-like wrinkled grains, which might be formed by the GO interaction with CS, as shown in Figure 2a. In the case of the CS-CuO composites (Figure 2b), the conglomerated grains are observed due to the strong interaction of CS with CuO particles. When the CUR is decorated on the CS-GO nanograins, the granular, irregular-shaped grains are observed with agglomeration for the CS-CUR-GO composite (Figure 2c). Figure 2d shows the SEM image of the CS-CUR-GO/CuO composite. The well-dispersed grains with inhomogeneous shapes and sizes of domains are observed. The prepared CS-CUR-GO/CuO composite consists of granular particles, along with bumped grains, which depict the enriched surface area and highly interacted grains in the resulting nanocomposites. Supplementary Materials Figure S2a–d shows the particle size analysis profiles of the CS-GO, CS-CuO, CS-CUR-GO, and CS-CUR-GO/CuO nanocomposites, respectively. The observed profile reveals the different sizes of nanoparticles in the prepared composites. The CS-CUR-GO/CuO hybrid composite exhibits a large number of grains exhibiting a 50 nm size. From the observation of various composite surfaces, the CS-CUR-GO/CuO hybrid composite provides enriched characteristics and a better morphological nature than the other intermediates.

**Figure 3:**
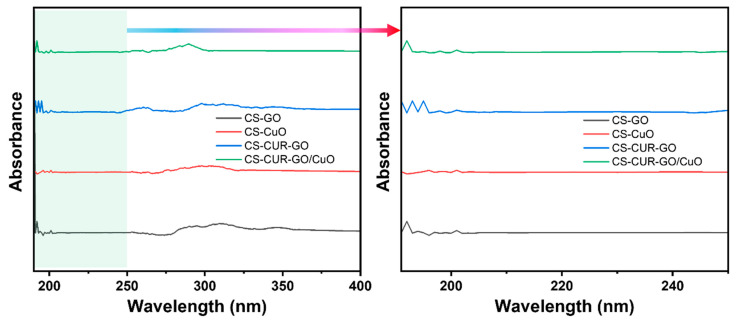
UV-Vis spectra and their magnified parts for CS-GO, CS-CuO, CS-CUR-GO, and CS-CUR-GO/CuO nanocomposites (Arrow indicates enlarged portion green highlighted zone).

The optical properties of the prepared composites were deliberately defined by the UV-Vis spectroscopy results. Figure 3 shows the UV-Vis spectral results of the prepared CS-GO, CS-CuO, CS-CUR-GO, and CS-CUR-GO/CuO nanocomposites between the range of 190 nm and 400 nm. The absorption profile reveals the peak between the 200 and 220 nm wavelengths owing to the existence of carbonyl groups in the resulting nanocomposites [54]. For CS-CuO, the absorption edge is slightly shifted to the blue region [55]. However, the shifting of the optical absorption edge towards the higher wavelength region is observed for the CS-CUR-GO nanocomposite. The observation of a red shift in the optical absorption edge is credited to the exhibiting of GO trapping in chitosan fibres. In the case of the CS-CUR-GO/CuO nanocomposite, the absorption edge is slightly moved towards the lower region due to the coexistence of CuO with CS-CUR-GO [56].

**Figure 4:**
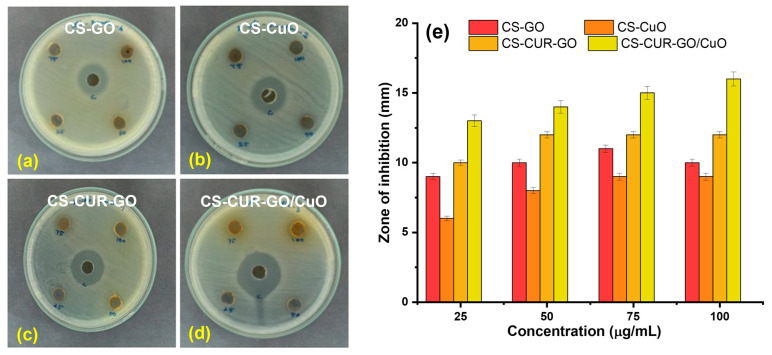
Antibacterial activities of (**a**) CS-GO, (**b**) CS-CuO, (**c**) CS-CUR-GO, and (**d**) CS-CUR-GO/CuO nanocomposites against Gram-negative *E. coli* bacterial organisms and (**e**) their zone of inhibition.

**Figure 5:**
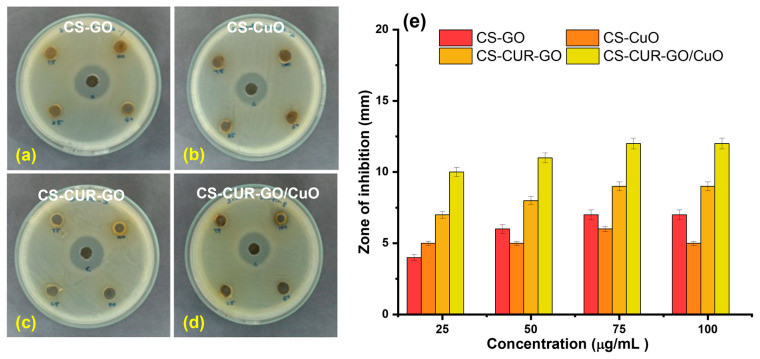
Antibacterial activities of (**a**) CS-GO, (**b**) CS-CuO, (**c**) CS-CUR-GO, and (**d**) CS-CUR-GO/CuO nanocomposites against Gram-positive *S. aureus* bacterial organisms and (**e**) their zone of inhibition.

The antibacterial studies of CS-GO, CS-CuO, CS-CUR-GO, and CS-CUR-GO/CuO were studied against *E. coli* and *S. aureus* pathogens. Several research works have proved the effective antibacterial activity of CUR and CS towards Gram-positive and Gram-negative pathogens [2,57]. Figure 4a–d show the antibacterial properties of CS-GO, CS-CuO, CS-CUR-GO, and CS-CUR-GO/CuO, respectively, against the Gram-negative *E. coli* pathogen by zone of inhibition assay. The enriched antibacterial activity is efficiently realized with an increase in concentrations for CS-CUR-GO/CuO. For the comparison, gentamycin was used as a control inhibitor for the antibacterial studies. The zone of inhibition of the CS-based nanocomposites versus *E. coli* is given in Figure 4e. Figure 5a–d show the zone of inhibition antibacterial properties of CS-GO, CS-CuO, CS-CUR-GO, and CS-CUR-GO/CuO, respectively, against the Gram-positive *S. aureus* pathogen. Similarly, the improved antibacterial activity against *S. aureus* is realized with an increase in concentration of the CS-CUR-GO/CuO composite. The zone of inhibition of the CS-based nanocomposites versus *S. aureus* is given in Figure 5e. The observation depicts the high zone of inhibition against *E. coli* and *S. aureus* pathogens by CS-CUR-GO/CuO among the various prepared composites. The multiple measurements established effective antibacterial properties of the prepared composites, which were defined by the standard deviations from the various results. Curcumin combats antibiotic resistance by restoring antimicrobial effectiveness to the derived composites. It also acts as a potent antiviral against viruses like feline infectious peritonitis [18]. Its antiviral properties arise from its ability to regulate multiple molecular targets involved in cellular events like transcriptional regulation and activation of cell signalling pathways, including programmed cell death and inflammatory processes, via intermolecular interactions [58]. It is more effective against Gram-positive bacteria than Gram-negative bacteria, with varying inhibiting properties depending on the species and strain. The susceptibility of a bacterial species is not related to their association with the genus and can vary greatly [15]. GO is an efficient antibacterial but tends to clump together, which limits its effectiveness. To improve GO’s antibacterial properties, it can be modified with CuO to increase its surface area and antibacterial properties [37]. A metal oxide form of CuO can give many more antibiotic and antibacterial properties when combined with GO. Some have antibacterial properties through the generation of ROS by shining light on their surface and can damage bacterial membranes by direct contact with their sharp edges [59].

Chitosan’s antibacterial effects involve interacting with peptidoglycan stabilization, altering the osmotic balance of the membrane wall and impacting the electron transport chain and oxygen reduction, thereby affecting the membrane’s energetic stability. Chitosan can interact with anionic structures on the surface of Gram-negative bacteria and directly with the cell wall layer of Gram-positive bacteria [60]. The results showed that the prepared hybrid composite CS-CUR-GO/CuO produced an improved zone of inhibition when compared with the other intermediates [34,39]. The composite CS-CUR-GO/CuO is better due to the presence of a natural antibiotic agent (curcumin) and other biomedical agents like chitosan, a metal oxide (CuO), and GO [61]. A probable structural mechanism for CS-CUR-GO/CuO is initially derived by the CuO nanoparticle-embedded GO core. Secondly, CUR successively encompassed the surface of CuO-GO nanoparticles to result in the chitosan-based nanocomplexes. In addition, the exterior surfaces of chitosan molecules can be cross-linked with each other via hydrogen bonding and hydrophobic force to produce complex microstructures [54].

**Figure 6:**
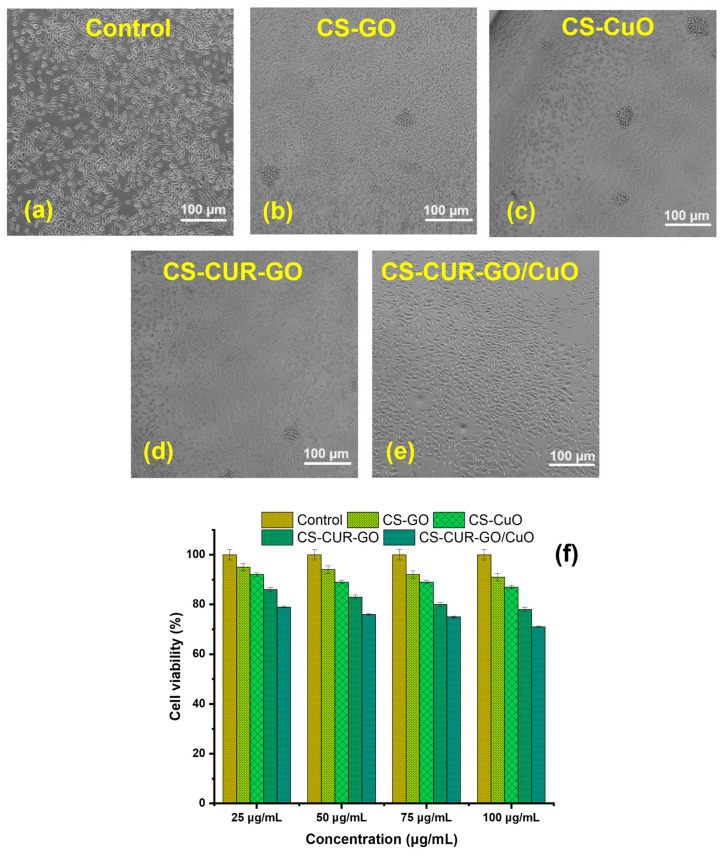
Cytotoxcity of mouse fibroblast cells at 24 h using (**a**) control, (**b**) CS-GO, (**c**) CS-CuO, (**d**) CS-CUR-GO, and (**e**) CS-CUR-GO/CuO hybrid nanocomposites and (**f**) their cell viability.

**Figure 7:**
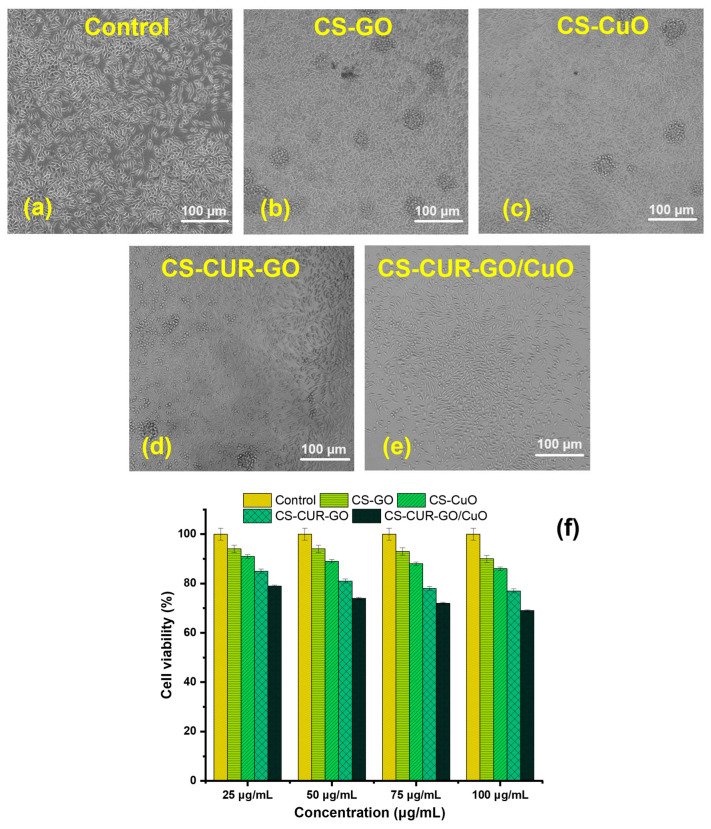
Cytotoxicity of mouse fibroblast cells at 48 h using (**a**) control, (**b**) CS-GO, (**c**) CS-CuO, (**d**) CS-CUR-GO, and (**e**) CS-CUR-GO/CuO hybrid nanocomposites and (**f**) their cell viability.

Figure 6 and Figure 7 illustrate the in vitro cytotoxicity of different concentrations (25, 50, 75, and 100 µg/mL) of control and CS-based composites on mouse fibroblast cell lines (L929) using the MTT test after 24 h and 48 h of exposure. Figure 6a–e show the morphological images for in vitro cytotoxicity of control, CS-GO, CS-CuO, CS-CUR-GO, and CS-CUR-GO/CuO, respectively, against fibroblast cells at 24 h. Figure 6f demonstrates the cell viability percentage after 24 h incubation with CS-based nanocomposites. Notably, at 24 h, the cytotoxicity of the CS-CUR-GO and CS-CUR-GO/CuO nanocomposites showed 93 and 89% cell viability, respectively, with 25 µg/mL. The cytotoxicity increases significantly with the increase in loading nanocomposite concentrations, as depicted in Figure 6f. The outcomes exhibit 84% and 79% vitality loss after the 24 h treatment with a 100 µg/mL concentration of CS-CUR-GO and CS-CUR-GO/CuO nanocomposites, respectively, indicating significantly high cytotoxicity against the fibroblast cell lines. Figure 7a–e show the morphological variations in control, CS-GO, CS-CuO, CS-CUR-GO, and CS-CUR-GO/CuO, respectively, against fibroblast cells at 24 h. The control cells (L929 cell lines) exhibited a regular structural organization, indicating non-cytotoxic effects or morphological alterations. The observed results revealed that the presence of GO and CuO in the hybrid nanocomposite formulations plays an immense role in realizing the improved cytotoxic nature. Figure 7f demonstrates the cell viability percentage after 24 h sensitization by the CS-based composites. Likewise, cell viability of 90%, 86%, 77%, and 69% were realized for fibroblast cells after 48 h of incubation by CS-GO, CS-CuO, CS-CUR-GO, and CS-CUR-GO/CuO at 100 µg/mL concentration, respectively. The findings underscore that the cytotoxicity of hybrid nanocomposites is intermixed with the concentrations and the internalization of particles into the cells. Additionally, the presence of CUR can sustainably enhance the anticancer effects, along with the metal oxide and chitosan complexes.

**Figure 8:**
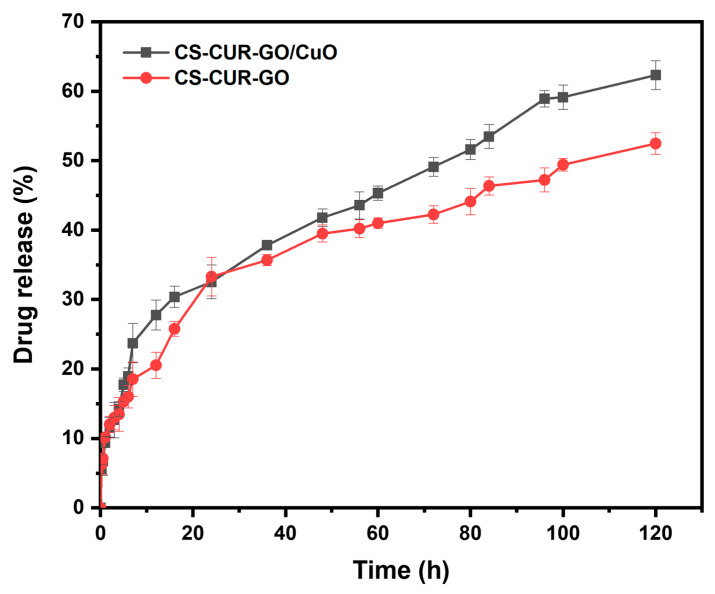
Drug release profile of curcumin from CS-CUR-GO and CS-CUR-GO/CuO nanocomposites in PBS at 37 °C.

Figure 8 depicts the drug-releasing effectiveness of CS-based nanocomposites. The loading effectiveness and drug release profile of CUR in PBS have been studied using the optimum settings. According to the findings, increasing the quantity of CS-CUR-GO/CuO nanocomposite allows the incremental loading and trapping of CUR with bulk-sized nanoparticles. The observation exhibits the zero-order kinetics of drug release for the prepared composites. Furthermore, larger curcumin concentrations limit the drug precipitation. Figure 8 indicates that CUR release is gradual and regulated at first, then becomes persistent. However, the release curves at 12 h show 21% and 28% increases from the CS-CUR-GO and CS-CUR-GO/CuO nanocomposites, respectively. In neutral pH, the delayed release causes a repulsive force between the positively charged CS polymer, causing it to swell. After 24 h, an equal proportion (33%) of drug diffused out of the CS-CUR-GO and CS-CUR-GO/CuO nanocomposites. On the contrary, an uneven diffusion rate was detected in the latter phase of CS-CUR-GO, whereas the CS-CUR-GO/CuO nanocomposite demonstrated considerable controlled and sustained drug release. Thus, the CS-CUR-GO/CuO nanocomposite with a release profile of 62% at 120 h is a promising nano-formulation for various controlled drug delivery applications.

## 3. Conclusions

A simple precipitation methodology was employed to form the CS-based hybrid composites. The prepared CS-GO, CS-CuO, CS-CUR-GO, and CS-CUR-GO/CuO nanocomposites were confirmed by the XRD and FT-IR studies. The optical properties were described using UV-Vis spectral analyses. The surface modification was captured using SEM micrographs for the CS-GO, CS-CuO, CS-CUR-GO, and CS-CUR-GO/CuO nanocomposites. The prepared composites were extensively used as antibacterial agents against *S. aureus* and *E. coli* bacteria. Also, the antibacterial activity of the CS-CUR-GO/CuO hybrid nanocomposite showed an extreme bactericidal effect against *S. aureus* and *E. coli* pathogens. The CS-CUR-GO/CuO nanocomposite could produce enriched cytotoxic behaviour for cultured mouse fibroblast cells. Thus, the resulting outcomes ascertained that blended chitosan and CUR hybrid nanocomposites offer rich biocompatibility and effective bacterial cell disruption functionalities as a potential candidate for antibacterial and cytotoxic applications. In the future, the derived nanocomposites will be used in vivo and in clinical studies to confirm their biocompatibility and practical uses.

## 4. Materials and Methods

### 4.1. Materials

All analytical grade chemicals, including chitosan (95%), graphite flakes, copper sulfate, hydrochloric acid (HCl), phosphoric acid (H_3_PO_4_), copper sulfate (CuSO_4_), sodium hydroxide (NaOH), ethanol, and nutrient agar, were acquired from Sigma Aldrich, Mumbai, India. Turmeric powder was purchased from the Agricultural University, Coimbatore. All the obtained chemicals were used as purchased without modifications for the experiments.

### 4.2. Preparation of Curcumin

Curcumin (CUR) was extracted from turmeric powder. A 30% aqueous solution of ethanol was mixed with 50 g of turmeric powder in a round-bottom flask for a couple of hours at 80 °C. CUR was extracted with the support of water reflux conditions, along with a free flow of water throughout the extraction process. A porcelain bit was incorporated for uniform and effective extraction.

### 4.3. Synthesis of Graphene Oxide

The graphene oxide (GO) was prepared by the modified Hummer’s route [13,62]. The acid mixture of H_2_SO_4_ and H_3_PO_4_ in the volume ratio of 9:1 was magnetically stirred for an hour. The procured graphite powder was blended into the mix and then magnetically stirred at ~75 °C for an hour. When the colour was significantly changed from black to grey–black, the mixture was mixed with potassium permanganate and subsequently stirred for six hours. Further, when the solution reached a dark brown colour, hydrogen peroxide was then mixed to reduce the permanganate, followed by magnetic stirring to realize a blackish brown colour. The mixture was then rinsed with 0.1 M HCl and de-ionized (DI) water for a short span of about 15 min. The result was vacuum-filtered and air-dried for a couple of hours and oven-dried at 200 °C for 30 min. The dried precipitates were ground with mortar and pestle to obtain a black-coloured, fine powder.

### 4.4. Synthesis of CS-CuO Nanocomposite

Briefly, 0.5 g of chitosan was dissolved in a mixture of 5 mL acetic acid and 20 mL of DI water and then subjected to magnetic stirring for 30 min. Additionally, 0.5 g of CuSO_4_ was mixed with 25 mL water to form a blue-coloured aqueous solution and then subjected to continuous magnetic stirring for 30 min. Further, the mixture was slowly mixed with 30% NaOH, along with the continuous stirring process, and then heated at 70 °C for 3 h. Finally, the precipitate was allowed to settle down, and then the precipitate was filtered and dried at 110 °C for 2 h. The dried precipitate was ground to form the fine CS-CuO powder.

### 4.5. Synthesis of CS-GO-Based Nanocomposites

Briefly, 0.5 g of chitosan was dissolved in a mixture of 5 mL acetic acid and 20 mL of DI water and then subjected to magnetic stirring for 30 min. Freshly prepared GO (0.5 g) was blended under continuous magnetic stirring with the above solution. Then, the solution was slowly mixed with 30% NaOH and moved to the hot surface at 70 °C. The collected precipitate was filtered and dried at 110 °C for 2 h. Finally, the CS-GO composites were obtained after grinding using mortar and pestle.

The commercially purchased chitosan was blended in the acetic acid–DI mixture under magnetic stirring. Then, freshly prepared GO water was mixed with the solution mixture, followed by the magnetic stirring. Then, the mixture solution was blended with 25 mL of CUR extract, followed by the slow addition of 30% NaOH, and moved to a hot surface at 70 °C. The collected precipitate was filtered and parched at 110 °C for 2 h. Ultimately, the ground fine powder of the CS-CUR-GO composite was obtained.

To form the CS-CUR-GO/CuO nanocomposite, a CuSO_4_ solution mixture was blended before the NaOH and then followed the same procedure. The collected precipitates were ground to form a fine powder, and the CS-CUR-GO/CuO composite was obtained.

### 4.6. Physiochemical Characterization 

The physicochemical properties of synthesized hybrid nanocomposites were determined by Nicolet 20 DXB Fourier transform infrared (FT-IR) spectrophotometer; Bruker (Billerica, MA, USA) D2 X-ray diffractometer (XRD); JEOL (Tokyo, Japan) JSM 6390 scanning electron microscope (SEM); Systronics 117 (Ahmedabad, India) UV-Vis NIR spectroscopy.

### 4.7. In Vitro Drug Release

To examine the CUR drug recovery, 250 mg of the CS-CUR-GO and CS-CUR-GO/CuO nanocomposites was directly dispersed in a fixed volume of phosphate-buffered saline (PBS, 10 mL) with pH 7.4 and located in a shaker-cum-incubator for 24 h at 37 °C at 200 rpm as per the previous literature [63]. The continuous release of CUR from the hybrid nanocomposites was monitored over a period of 120 h. At every regular interval of time, 1 mL of the sample solution was collected and restored with 1 mL of freshly equipped PBS solution at an appropriate pH. To determine the CUR release, the collected samples were centrifuged (>8500 rpm) and the final supernatant was used for absorbance using a UV-Vis spectrophotometer to determine the concentration of unreleased CUR by using a standard curve of CUR. The drug release % was calculated by using the following Equation (1):

(1) $$In\;vitro\;drug\;release\;(\%)=\frac{Released\;composites\times 100}{Total\;composites}$$

### 4.8. Antibacterial Studies

The disk diffusion method is an established laboratory technique to test the effectiveness of antibiotics or antiseptics on microorganisms [2,64]. The prepared composite-specific concentration (25–100 µg/mL) contained in a filter-paper disc was placed onto an agar plate inoculated with *S. aureus* or *E. coli* pathogens. The prepared composites were diffused out of the disc and into the agar, making a concentration gradient. Next, inoculum plates were grown at 37 °C for 24 h in an ambient environment. Further, the diameter of the inhibiting zone around each disc was measured.

### 4.9. Cytotoxicity Studies

In 96-well growth plates, the L929 cell line (procured from ATCC, Pune, India) growth was performed by Dulbecco’s customized minimum essential medium (DMEM) accompanied by 10% fetal bovine serum (FBS) and antibiotics (penicillin and streptomycin) [65,66]. The cytotoxicity of the CS-based hybrid nanocomposites was investigated using the standard MTT (3-(4,5-Dimethylthiazol-2-yl)-2,5-diphenyltetrazolium bromide) assay. L929 cells were planted in a 96-well plate with a density of 10,000 cells/well and grown in normal circumstances in a CO_2_ incubator. For the dilution with the medium, distinct concentrations of the CS-based composites (25, 50, 75, and 100 µg/mL) were used. After attaining 90% confluence, the cells were cured with the various nanocomposites (100 mL) for 24 and 48 h. Untreated cells in conditions devoid of nanocomposites were employed as a negative control (i.e., 100% viable). MTT reagent was applied to each well four hours before the conclusion of the experiment and incubated at 37 °C in a CO_2_ incubator, followed by a one-hour incubation with a solubilization buffer. Finally, the solution optical density was measured at 570 nm, and cell viability (%) was estimated. For each sample, triplicate experiments were performed to confirm the results. All the biological experiments were repeated in three experiments, and their mean values were presented with the standard deviation results.

## Data Availability

The data presented in this study are available on request from the corresponding author. The data are not publicly available due to privacy or ethical restrictions.

## Supplementary Materials

The following supporting information can be downloaded at: https://www.mdpi.com/article/10.3390/antibiotics13070620/s1, Figure S1 XRD spectra of CS and CUR; Figure S2 Particle size analysis profiles for the prepared composites.
