# Supplementary material for: Chitosan-Integrated Curcumin–Graphene Oxide/Copper Oxide Hybrid Nanocomposites for Antibacterial and Cytotoxicity Applications
Source: Antibiotics (Basel). 2024 Jul 3;13(7):620. doi: 10.3390/antibiotics13070620 (PMC11273410; doi:10.3390/antibiotics13070620)
Supplement: Supplementary file 1 [file antibiotics-13-00620-s001.zip › antibiotics-3077481-supplementary.pdf]

Supplementary Materials

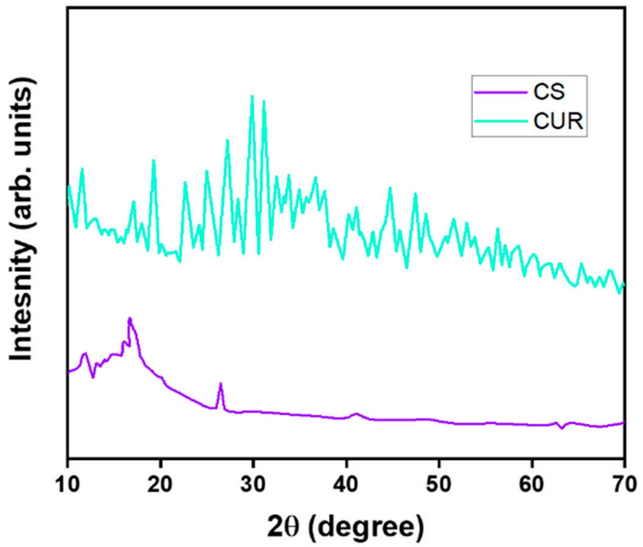

Figure S1. XRD spectra of CS and CUR.

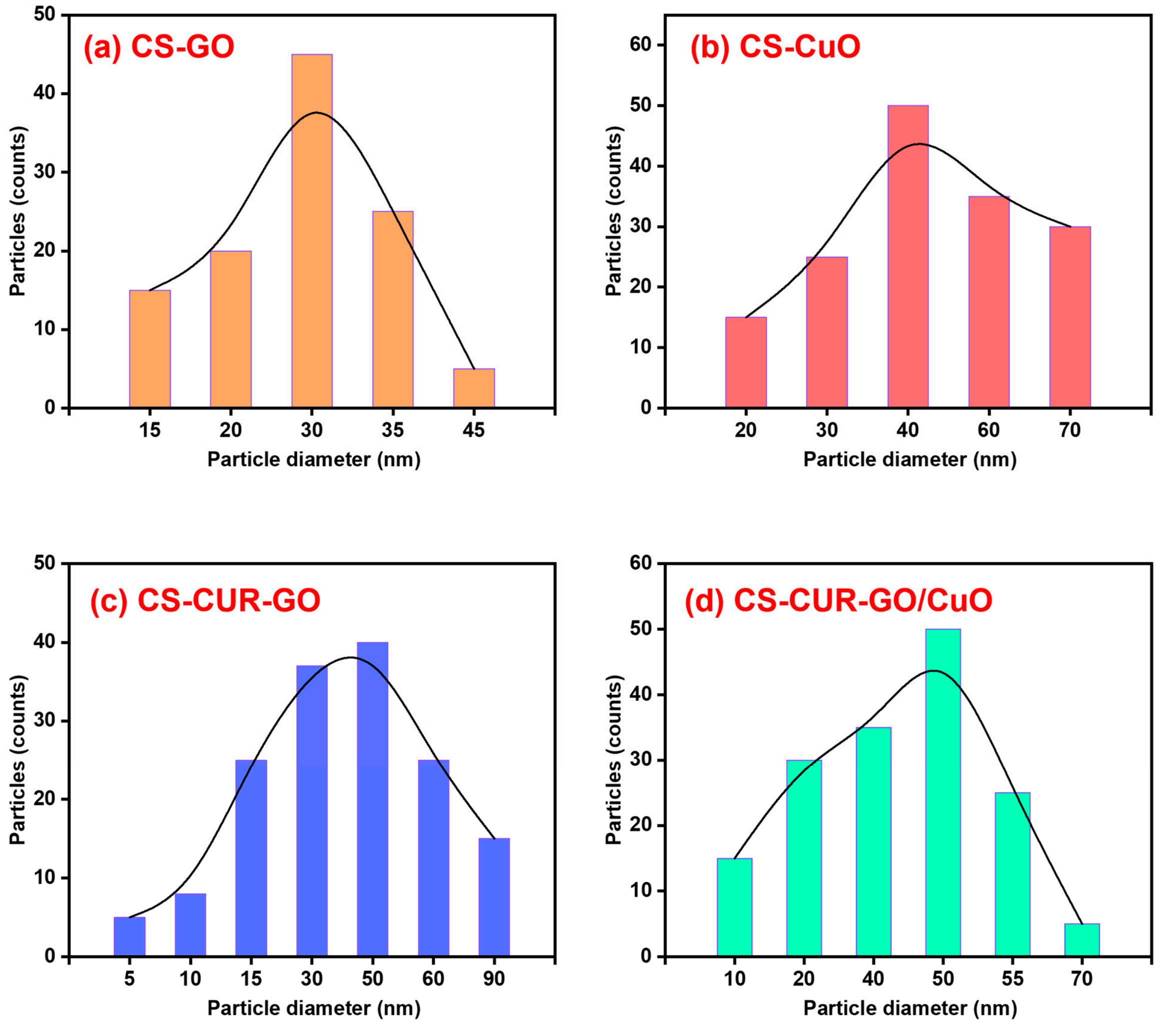

Figure S2. Particle size analysis profiles for the prepared composites (**a**) CS-GO, (**b**) CS-CuO, (**c**) CS-CUR-GO and (**d**) CS-CUR-GO/CuO.
